# Proton pump inhibitor use and the risk for Parkinson’s disease: A nationwide population-based study in Taiwan

**DOI:** 10.1097/MD.0000000000033711

**Published:** 2023-05-12

**Authors:** Huan-Lin Chen, Wei-Yi Lei, Jen-Hung Wang, Ming-Jong Bair, Chien-Lin Chen

**Affiliations:** a Division of Gastroenterology, Department of Internal Medicine, Taitung MacKay Memorial Hospital, Taitung, Taiwan; b Mackay Medical College, New Taipei, Taiwan; c Division of Gastroenterology, Department of Internal Medicine, Hualien Tzu Chi Hospital, Buddhist Tzu Chi Medical Foundation, Hualien, Taiwan; d Department of Medical Research, Hualien Tzu Chi Hospital, Buddhist Tzu Chi Medical Foundation, Hualien, Taiwan; e Institute of Medical Sciences, Tzu Chi University, Hualien, Taiwan.

**Keywords:** gastric acid-related disorders, nationwide population-based study, Parkinson’s disease, proton pump inhibitors

## Abstract

Previous studies have shown that proton pump inhibitors (PPIs) are associated with an increased risk of dementia. However, little is known about the relationship between PPIs use and Parkinson’s disease (PD). This study aimed to examine whether PPI use was associated with an increased risk of developing clinically verified PD. This used data from the Taiwan National Health Insurance Research Database for the period between 1999 and 2011, and patients with PPI use were compared with 1 to 1 propensity score-matched controls by age, sex, cohort entry year, and comorbidity. A multivariate analysis was performed using Cox proportional hazards models to estimate the association between PPI use and PD risk. Subgroup analyses according to sex, age, and comorbidities were also conducted. In total, 56,785 PPI users and 56,785 matched controls were enrolled in this study. In the PPI cohort, 366 patients developed PD during a median follow-up of 5.0 years. The incidence rate of PD was 1.48-fold higher in PPI users than in non-PPI users (90.0 vs 133.2 per 100,000 person-years), with an adjusted hazard ratio of 1.76 (95% confidence interval, 1.48–2.08). In the subgroup analysis, the adjusted risk of PD in the PPI and non-PPI cohorts increased in the subgroups regardless of age, sex, and comorbidities. The results of this retrospective, nationwide, population-based cohort study in Taiwan indicate that PPI use is associated with the risk of PD development. Further mechanistic studies on the effect of PPI on PD are needed.

## 1. Introduction

Parkinson’s disease (PD) is a common neurodegenerative disease, especially in the elderly population. It is characterized by a progressive loss of dopamine-producing substantia nigra cells, resulting in a broad spectrum of motor and non-motor features. Common symptoms include tremors, muscle rigidity, slow physical movement, autonomic impairment, and gastrointestinal dysfunction.^[1]^

Since the 1960s, studies^[2,3]^ have revealed that gastric ulcer disease is associated with PD. In addition, *Helicobacter pylori (HP*) infection has been implicated in the development of parkinsonism, which may be related to familial aggregation and association with water sources.^[4,5]^ In another study,^[6]^ patients with PD were 3 times more likely to test positive for *HP* serology than controls, and their siblings had *HP* seropositivity and signs of Parkinsonism. These diseases (gastric ulcers and *HP* infections) are treated with proton pump inhibitors (PPIs).

PPIs are commonly used for the treatment of gastric acid-related disorders such as gastroesophageal reflux and peptic ulcer disease. The main mechanism is irreversible inhibition of the H+/K+-ATPase pump, which decreases gastric acid production. PPIs have excellent treatment efficiency and safety profile; thus, they have become one of the most prescribed drugs worldwide. They are also effective in treating ulcers associated with the use of nonsteroidal anti-inflammatory drugs. Thus, until now, they have been useful prophylactic treatments for patients taking nonsteroidal anti-inflammatory drugs and low-dose aspirin.^[7–11]^

PPIs are generally considered to be effective and safe. However, long-term PPI use has been associated with an increased risk of adverse events,^[12–16]^ such as hypomagnesemia, kidney diseases, bone fractures, and even microscopic colitis. Given these adverse effects, their safety and role in cognitive function, including the risk of developing dementia and Alzheimer’s disease (AD), have been questioned. Several studies^[17–19]^ have described an association between PPI use and a greater risk of developing dementia and AD, particularly in older people. However, the relationship between PPI use and PD is not well understood. Thus, in this study, we aimed to provide real-world data on PPI use and related changes over the past decade in the entire Taiwanese population to examine whether PPI use is associated with an increased risk of developing clinically verified PD.

## 2. Materials and methods

### 2.1. Data source

The Taiwan National Health Insurance program was established in 1995; by 2010, it had covered more than 23 million residents. This system represented nearly the entire population of Taiwan (99%). Complete patient health information, including sociodemographic data, medical procedures, drug prescriptions, and diagnostic codes based on the International Classification of Diseases, 9th revision, Clinical Modification codes, were recorded in the National Health Insurance Research Database (NHIRD). In 2000, the National Health Research Institute released a cohort dataset comprising 1,000,000 randomly sampled individuals. The study sample was retrieved from the Longitudinal Health Insurance Database 2000 (LHID 2000). The data source was encrypted to protect patient privacy. All the data were extracted anonymously.

### 2.2. Ethics statement

The Ethics Committee of Tzu Chi Medical Center, Taiwan approved the study protocol, which conformed to the provisions of the Declaration of Helsinki in 1995 (as revised in Edinburgh 2000). Written informed consent was obtained from all the participants. Additionally, patient anonymity was preserved. This study was approved by the Research Ethics Committee of the Hualien Tzu Chi Hospital, Buddhist Tzu Chi Medical Foundation, Hualien, Taiwan.

### 2.3. Study design and participants

This was an observational drug-utilization study of PPI use among Taiwanese adults (aged ≥ 20 years) between January 1, 1999, and December 31, 2011. Data were obtained from the NHIRD in Taiwan. Accordingly, we conducted a retrospective cohort study of PPI users who were compared with 1 to 1 propensity score-matched controls according to age, sex, cohort entry year, and comorbidity. Multivariate analysis was performed using Cox proportional hazards models to estimate the association between PPI use and PD risk. Subgroup analyses (stratified by sex, age, and comorbidities) were also performed.

**Figure 1.**
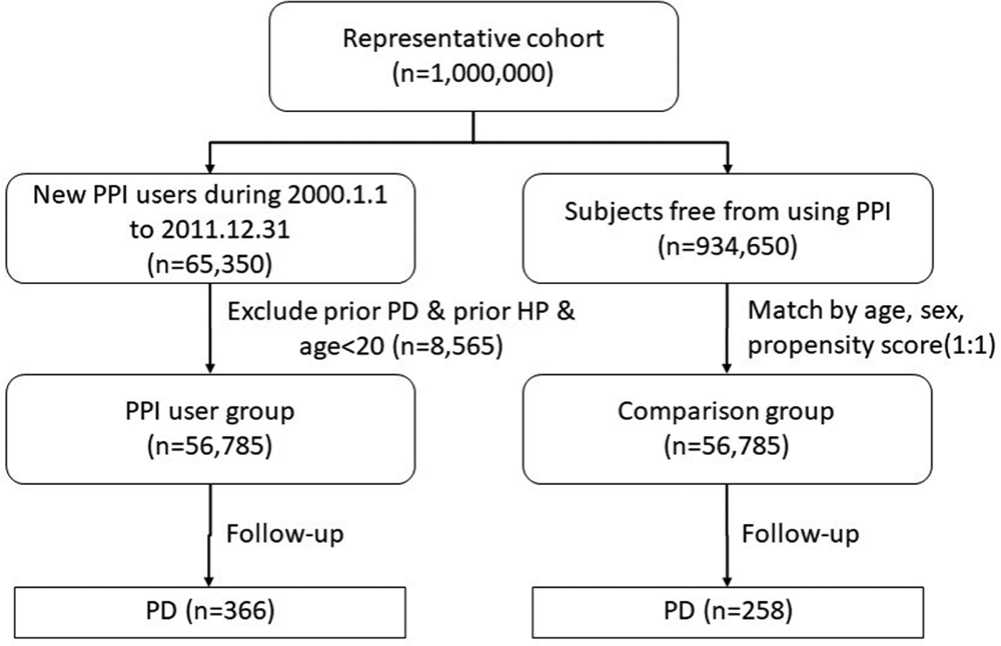
Flowchart of subjects enrolled into the study.

We enrolled 65,350 new PPI users in the LHID 2000 and excluded patients with PD, previous *HP* infection, and age < 20 years. Finally, 56,785 patients were included in this study. PPI users who were matched to non-PPI users by age, sex, comorbidities, and cohort entry year were selected for comparison. Comorbidities included preexisting cirrhosis, diabetes mellitus, chronic kidney disease, chronic obstructive pulmonary disease, hypertension, coronary artery disease, epilepsy, and cerebrovascular disease. We also used an outpatient pharmacy prescription database to identify drug dosages, types, prescription dates, total number of pills dispensed, and supply days. We then summarized PPI use (ATC code: A02BC). The flowchart of the selection process is shown in Figure 1.

### 2.4. Statistical analysis

We performed chi-square and independent t-tests to assess between-cohort differences in frequencies or means of variables. Cohen d was also used to evaluate the effect size. Since PPI use and PD risk were the main interest in this study, each participant was followed up to accumulate person-time from the index date to new-onset PD in 5 years. If the participants died before PD onset, they were censored to account for competing risks, which could be attributed to other causes. All cases with no endpoints occurring during follow-up were also investigated. We used the Kaplan–Meier curve and log-rank test to estimate and compare the incidence rates of PD after matching for age, sex, and propensity scores. We also used a Cox proportional hazard model to estimate the hazard ratio (HR) and accompanying 95% confidence interval (CI) of PD with adjustment for confounders. Statistical significance was set significant. SAS (version 9.4; SAS Institute, Inc., Cary, NC) and SPSS version 17 (SPSS Inc., Chicago, IL) were used for data analysis.

## 3. Results

### 3.1. Participant characteristics

The demographic characteristics of the participants are presented in Table 1. From the LHID 2000, 56,785 patients were new PPI users (mean age, 47.1 years; standard deviation, 14.9). The control group included 56,785 participants matched for age and comorbidities. The majority of the patients in both cohorts were men (51.4%). The most common comorbidities were hypertension, diabetes mellitus, and cirrhosis. No significant differences in age, sex, or baseline comorbidities were found between the 2 groups.

**Table 1:** Demographic data on patients with and without PPI Usage.

Variables	PPI user (n = 56,785)		Control (n = 56,785)	
Age	47.1 ± 14.9		47.0 ± 14.7	
Age group				
<60 yr/o	46,396	81.7%	46,390	81.7%
≥60 yr/o	10,389	18.3%	10,395	18.3%
Gender				
Male	29,192	51.4%	29,061	51.2%
Female	27,593	48.6%	27,724	48.8%
Cirrhosis	3885	6.8%	3875	6.8%
Hypertension	10,215	18.0%	10,651	18.8%
Diabetes	5045	8.9%	5299	9.3%
CKD	815	1.4%	596	1.0%
CAD	3051	5.4%	3160	5.6%
COPD	1939	3.4%	2009	3.5%
Epilepsy	227	0.4%	244	0.4%
CVD	1744	3.1%	1818	3.2%
Follow-up yr	4.84 ± 3.24		5.05 ± 3.21	

Data are presented as n and percentage.

### 3.2. Incidence of PD

In total, 624 (0.5%) participants were diagnosed with PD over a mean follow-up period of 5 years. The most significant risk factor was age, which is consistent with a previous report. Participants aged > 60 years had a significantly higher risk of developing PD than those aged < 60 years (adjusted HR = 11.46). Further data analysis showed that PPI users also had a significantly higher probability of developing PD than non-PPI users (adjusted HR = 1.76). Moreover, patients with comorbidities, such as hypertension, cirrhosis, chronic kidney disease, coronary artery disease, chronic obstructive pulmonary disease, and cerebrovascular disease, had an increased risk of PD development (Table 2).

**Table 2:** Independent predictors of new-onset PD.

Variables	No. of PD (%)	Hazard ratio† (95% CI)			
		Crude	*P* value	Adjusted	*P* value
Age group					
<60 yr/o	152 (0.16%)	1.00		1.00	
≥60 yr/o	472 (2.27%)	16.49 (13.73–19.80)	<.001*	11.46 (9.33–14.09)	<.001*
Gender	0.16%				
Male	297 (0.51%)	1.00		1.00	
Female	327 (0.59%)	1.20 (1.03–1.41)	.022*	1.04 (0.89–1.22)	.637
PPI User					
No	258 (0.45%)	1.00		1.00	
Yes	366 (0.64%)	1.48 (1.26–1.74)	<.001*	1.76 (1.48–2.08)	<.001*
Cirrhosis					
No	563 (0.53%)	1.00		1.00	
Yes	61 (0.79%)	1.48 (1.14–1.93)	.004*	1.31 (1.00–1.71)	.046*
Hypertension					
No	301 (0.32%)	1.00		1.00	
Yes	323 (1.55%)	5.77 (4.93–6.76)	<.001*	1.66 (1.39–1.98)	<.001*
Diabetes					
No	485 (0.47%)	1.00		1.00	
Yes	139 (1.34%)	3.54 (2.93–4.28)	<.001*	1.21 (0.99–1.47)	.064
CKD					
No	604 (0.54%)	1.00		1.00	
Yes	20 (1.42%)	3.74 (2.39–5.84)	<.001*	1.71 (1.09–2.68)	.019*
CAD					
No	506 (0.47%)	1.00		1.00	
Yes	118 (1.90%)	4.49 (3.68–5.49)	<.001*	1.31 (1.06–1.61)	.012*
COPD					
No	551 (0.50%)	1.00		1.00	
Yes	73 (1.85%)	3.99 (3.13–5.09)	<.001*	1.46 (1.14–1.88)	.003*
Epilepsy					
No	620 (0.55%)	1.00		1.00	
Yes	4 (0.85%)	1.76 (0.66–4.71)	.258	1.23 (0.46–3.33)	.678
CVD					
No	538 (0.49%)	1		1	
Yes	86 (2.41%)	6.12 (4.87–7.68)	<.001*	1.62 (1.28–2.06)	<.001*

† Cox proportional hazards model.

cDDDs = cumulative defined daily doses, CI = Confidence Interval, PD = Parkinson’s disease.

*P value < .05 was considered statistically significant after test.

**Figure 2.**
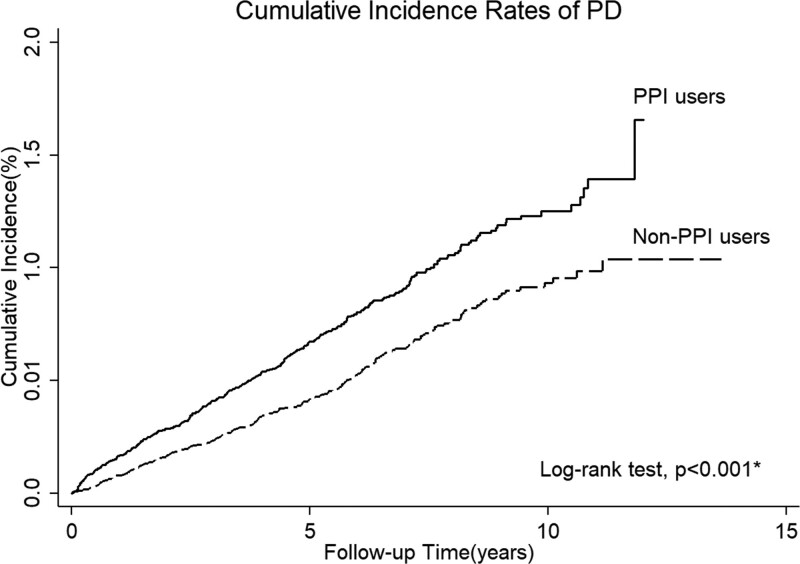
Kaplan–Meier curves showing a significant difference in cumulative incidence of PPI user among patients with PD and controls. PD = Parkinson’s disease.

A total of 366 PPI users (0.64%) and 258 controls (0.45%) developed PD during the follow-up period. In the log-rank test, PPI users had a significantly higher incidence of PD than non-PPI users (*P* *< *.001) (Fig. 2). In the Cox proportional hazard model analysis, PPI use was independently associated with an increased risk of developing PD (HR = 1.76; *P* *< *.001, Table 3).

**Table 3:** Independent predictors of new-onset PD.

Variable	All patients (n = 113,570)		PPI user (n = 56,785)		Control (n = 56,785)	
PD						
Yes	624	0.5%	366	0.6%	258	0.5%
No	112,946	99.5%	56,419	99.4%	56,527	99.5%
Crude HR (95% CI)			1.48	(1.26–1.74)	1.00	
Adjusted HR (95% CI)			1.76	(1.48–2.08)	1.00	

Adjusted for age, sex, H2 Blockers dosage, and comorbidities.

CI = confidence interval, HR = hazard ratio, PD = Parkinson’s disease, PD = Parkinson’s disease, PPI = proton pump inhibitor.

In the subgroup analyses based on age, sex, and comorbidities to evaluate PD risk (Table 4), PPI use was significantly positively associated with PD development, except for coronary artery disease (HR = 1.88; 95% CI 1.43 ± 2.48, *P* = .851) and chronic obstructive pulmonary disease (HR = 1.69; 95% CI 1.21 ± 2.36, *P* = .822). We further investigated the PD group according to PPI dosage and compared it with the control group. A total of 366 PPI users received different dosage regimens and 259 were non-PPI users. PPI users tended to be at risk for PD development compared with non-PPI users, even those who were prescribed a low PPI dosage (HR = 2.32; 95% CI 1.90 ± 2.82, *P* < .001) (Table 5).

**Table 4:** Adjusted hazard ratios of PD associatd PPI usage interaction with comorbidity.

Variable		N	Event	Adjusted* HR (95% CI)
PPI use	Cirrhosis			
No	No	52,910	229	Reference
No	Yes	3875	29	1.44 (0.98–2.12)
Yes	No	52,900	334	1.54 (1.30–1.82)
Yes	Yes	3885	32	2.05 (1.42–2.97)
PPI use	Hypertension			
No	No	46,134	124	Reference
No	Yes	10,651	134	1.46 (1.13–1.88)
Yes	No	46,570	177	1.52 (1.21–1.91)
Yes	Yes	10,215	189	2.24 (1.76–2.85)
PPI use	Diabetes			
No	No	51,486	205	Reference
No	Yes	5299	53	1.23 (0.91–1.67)
Yes	No	51,740	280	1.45 (1.21–1.74)
Yes	Yes	5045	86	2.24 (1.74–2.89)
PPI use	CKD			
No	No	56,189	252	Reference
No	Yes	596	6	1.52 (0.68–3.42)
Yes	No	55,970	352	1.51 (1.28–1.77)
Yes	Yes	815	14	2.82 (1.64–4.84)
PPI use	CAD			
No	No	53,625	210	Reference
No	Yes	3160	48	1.28 (0.93–1.76)
Yes	No	53,734	296	1.53 (1.28–1.83)
Yes	Yes	3051	70	1.88 (1.43–2.48)
PPI use	COPD			
No	No	54,776	228	Reference
No	Yes	2009	30	1.17 (0.80–1.72)
Yes	No	54,846	323	1.53 (1.29–1.81)
Yes	Yes	1939	43	1.69 (1.21–2.36)
PPI use	Epilepsy			
No	No	56,541	257	Reference
No	Yes	244	1	1.03 (0.14–7.31)
Yes	No	56,558	363	1.52 (1.29–1.78)
Yes	Yes	227	3	2.97 (0.95–9.28)
PPI use	CVD			
No	No	54,967	219	Reference
No	Yes	1818	39	1.74 (1.23–2.46)
Yes	No	55,041	319	1.58 (1.33–1.88)
Yes	Yes	1744	47	2.03 (1.47–2.81)

HR = hazard ratio, CI = confidence interval, PD = Parkinson’s disease.

* model adjusted with age and gender.

**Table 5:** Independent predictors of new-onset PD in patients with different PPI dosage.

Variable	No. of patients	PD (%)	Crude HR (95% CI)	*P* value	Adjusted HR (95% CI)	*P* value
PPI non-user	56,785	258 (0.45%)	1			
PPI cDDDs < 90	26,166	175 (0.67%)	1.59 (1.31, 1.92)	<.001*	2.32 (1.90, 2.82)	<.001*
PPI cDDDs 90–180	18,014	100 (0.56%)	1.37 (1.08, 1.72)	.008*	1.54 (1.22, 1.96)	<.001*
PPI cDDDs ≥ 180	12,605	91 (0.72%)	1.43 (1.12, 1.81)	.004*	1.30 (1.01, 1.68	.042*

Adjusted for age, sex, H2 Blockers dosage, and comorbidities.

cDDDs = cumulative defined daily doses, CI = confidence interval, HR = hazard ratio, PD = Parkinson’s disease, PPIs = proton pump inhibitors.

*P value < .05 was considered statistically significant after test.

## 4. Discussion

PD is the second most common neurodegenerative disease, with a median age-standardized annual prevalence of 14 per 100,000 people in high-income countries. It is also an age-related disorder,^[20]^ occurring in 160 of 100,000 people aged ≥ 65 years. The same data were obtained in the present study, in which participants aged > 60 years had a significantly higher probability of developing PD than those aged < 60 years did. However, the exact cause of PD remains unknown, even with precision instruments. Several studies have reported that both inherited and environmental factors may play a role in PD development.^[5,21]^ Participants with a family member with PD had an increased risk of developing PD, revealing certain genes as inheritable risk factors. Other risk factors include exposure to certain pesticides or prior head injuries;^[22,23]^ however, none of these have been conclusively proven.

Recent studies have suggested that patients receiving PPIs are at a significantly increased risk of developing dementia and AD.^[17–19]^ In our population-based case–control study, PPI use was positively associated with PD risk, which was also a promoting factor for PD risk in patients with comorbidities. The incidence was 0.6%, and PPI use increased the prevalence of PD by 1.76 times than that of age-matched controls. To the best of our knowledge, this is the largest population-based study to examine PPI use as a risk factor for PD development using a matched cohort design and long-term follow-up period.

PPIs have a very good safety profile and excellent treatment effects for short-term treatment of acid-related disorders. However, the effects of long-term PPI administration remain unclear. In previous studies, long-term PPI use has been associated with an increased risk of adverse events, such as vitamin B12 deficiency, iron-deficiency, hypomagnesemia, and intestinal infections,^[12–16]^ which may aggravate the risk of PD.

In humans, vitamin B12 is released from food proteins following peptic digestion in highly acidic environments. Vitamin B12 is involved in the conversion of methylmalonyl coenzyme A to succinyl coenzyme A and the production of tetrahydrofolate and methionine. They are important molecules for methylation reactions in the nervous system.^[24]^ The amount of vitamin B12 may decrease with PPI use. In deficiency states, serum levels of homocysteine (Hcy) and methylmalonic acid (MMA) increase. There has been a great deal of speculation regarding the neurotoxicity of Hcy in dopaminergic cells, which may contribute to PD progression. In addition, animal experiments have revealed that MMA causes striatal degeneration when injected directly into rat brain.^[25]^ A randomized study^[26]^ analyzed 58 participants from a PD database and compared them with age and sex-matched controls. In this study, neuropathy markedly increased in the PD group compared to that in the control group (55% vs 9%). Regarding laboratory data, lower vitamin B12 levels and higher MMA and Hcy serum levels were noted in patients with PD with neuropathy. Therefore, long-term PPI use should be prescribed carefully, especially in older patients. Alternatively, vitamin B12 supplementation may be advised for all patients with PD as no adverse effects of vitamin B12 excess have been reported.

Gastric acid facilitates the absorption of iron salts in food and conventional iron-containing medicines. Sarzynski et al^[27]^ and Ajmera et al^[28]^ recommended considering the possibility of reduced iron absorption and anemia in older patients. In a population-based cohort study that investigated PD risk in Taiwan, patients with iron-deficiency anemia tended to exhibit a higher PD risk (aHR, 1.49; 95% CI 1.24–1.79; *P* < .001).^[29]^ Iron-deficiency impairs erythropoiesis and enhances erythropoiesis, resulting in anemia, whereas increased iron deposition is noted in the substantia nigra of patients with PD. In addition, iron-deficiency impairs dopamine synthesis and reuptake,^[30,31]^ which are related to motor symptoms in patients with PD.

Magnesium deficiency is an undesirable effect of continuous, at least 1 year. The decline occurs gradually after the discontinuation of PPI (normalization for 1–2 weeks), but with PPI reintroduction, the deficit manifests again. Human studies investigating the potential role of Mg in PD are limited. The most recently published study was a multicenter hospital-based case–control study in Japan that examined dietary intake of metals in patients who were found to be within 6 years of PD onset.^[32]^ This study found that higher magnesium concentrations were associated with a reduced risk of PD. Thus, magnesium supplementation may be considered in patients with prolonged PPI treatment.

Gastric acid destroys many bacteria entering the stomach, and the suppression of the gastric acid barrier with PPIs may cause several problems, such as overgrowth of bacteria in the stomach and duodenal fluids. An Italy 450-patient study using a glucose hydrogen breath test revealed that small intestinal bacterial overgrowth (SIBO) occurred significantly more frequently among long-term PPI users.^[33]^ SIBO might affect the progression of PD through the induction of a peripheral immune response, disruption of the blood–brain barrier, and mediation of neuroinflammation.^[34,35]^ SIBO might create a proinflammatory environment, as reported in patients newly diagnosed with PD and supported by a study that quantified local inflammation and enteric glial reactions in gastrointestinal biopsies of patients with PD.^[36,37]^ In addition, SIBO might impair drug absorption following inflammation or partial metabolism of levodopa and might contribute to reduced gastric emptying through inflammatory effects on entero-chromaffin-like cells, which may play a role in the pathophysiological changes associated with motor fluctuations.^[38]^

The majority of adverse effects of PPIs are conflicting, and their clinical effects are not large enough. Since most studies concerning the adverse events of PPIs are retrospective and observational, potential biases in inclusion are not negligible. Under these conditions, only study results showing a large clinical effect can be reliable and clinically important. Therefore, the clinically meaningful risk of long-term PPI administration has not yet been fully established. However, even under these conditions, gastroenterologists should carefully balance the merits and demerits of long-term PPI administration in daily clinical practice.

## 5. Conclusion

We analyzed a large longitudinal Taiwan NHIRD between 1999 and 2011 and confirmed that long-term PPI use was associated with an increased risk of incident PD, especially in the older population. Thus, the avoidance of prolonged PPI therapy may contribute to PD prevention. This study could only provide a statistical association between PPI use and PD risk. The possible underlying biological mechanisms should be explored in future studies. To evaluate and establish direct cause–effect relationships between PPI use and incident PD in older people, randomized, prospective clinical trials are needed.

## Acknowledgments

This study was supported by a grant, TCRD 103–36, from Hualien Tzu Chi Hospital, Buddhist Tzu Chi Medical Foundation, Hualien, Taiwan.

## Author contributions

**Data curation:** Ming-Jong Bair, Chien-Lin Chen.

**Formal analysis:** Jen-Hung Wang.

**Investigation:** Wei-Yi Lei, Ming-Jong Bair, Chien-Lin Chen.

**Methodology:** Wei-Yi Lei, Chien-Lin Chen.

**Project administration:** Wei-Yi Lei, Chien-Lin Chen.

**Software:** Jen-Hung Wang.

**Writing – original draft:** Huan Lin Chen.

**Writing – review & editing:** Wei-Yi Lei.
